# Spatial relationship between carbon emissions and ecosystem service value based on land use: A case study of the Yellow River Basin

**DOI:** 10.1371/journal.pone.0318855

**Published:** 2025-02-21

**Authors:** Gubu Muga, Damien S. Tiando, Chong Liu

**Affiliations:** 1 College of Economics and Management, Mianyang Teachers’College, Mianyang, China; 2 School of Public Administration, China University of Geosciences, Wuhan, P. R. China; 3 China–Africa Institute (Wuhan), China University of Geosciences, Wuhan, P. R. China; 4 Anhui Jianzhu University, School Public Policy & Management, Hefei, P. R. China; Tongji University School of Economics and Management, CHINA

## Abstract

Land use changes significantly impact both carbon emissions and ecosystem service value (ESV). However, few studies have been conducted on the spatial relationship between land use carbon emissions (LUCE) and ESV. Thus, focused on the Yellow River Basin (YRB), this study independently calculates carbon emissions from land use change (LUCE) and ecosystem service values (ESV) in the region. Utilizing spatial autocorrelation methods, we analyze the spatiotemporal pattern of LUCE and ESV and subsequently apply the bivariate spatial autocorrelation method to explore their spatial relationship. The results prove that: (1) The YRB’s LUCE has continuously increased, with construction land acting as the dominant carbon source and woodland acting as the main carbon sink. The LUCE in the YRB had a positive spatial autocorrelation. (2) The YRB’s ESV increased. Spatially, the ESV in the YRB showed a positive autocorrelation. (3) Both LUCE and ESV exhibited negative spatial autocorrelation, with predominant patterns of bivariate localized spatial autocorrelation identified as High-Low agglomeration (H-L) and Low-High agglomeration (L-H). Cities with the L-H pattern were primarily located in Qinghai Province and Inner Mongolia. In contrast, cities with the H-L pattern were mainly observed in the western section of Shandong and the northeastern region of Henan. The study revealed the negative impact of increased carbon emissions from land use on the value of ecosystem services, providing assistance in the development of relevant environmental policies and promoting sustainable development in the YRB.

## 1. Introduction

Global warming has emerged as the most pressing threat confronting civilization during the Anthropocene epoch. Our global objective stated in the Paris Agreement of 2015 is to reduce the average increase in global temperatures over this century to under 2 degrees Celsius and well below a 1.5 degree Celsius increase over levels before industrialization, with a maximum limit of 3 degrees Celsius [1,2]. Land use change occurs through the dynamic interaction of natural environmental processes and human actions in specific geographical and temporal settings [3,4]. Changes in land use driven by anthropogenic actions, together with energy consumption patterns, are widely known as the important drivers of worldwide warming [5]. While energy usage associated with construction land has an indirect effect on regional carbon emissions, land use change has a direct influence on carbon stocks by altering ecosystem types [5]. Intergovernmental Panel on Climate Change (IPCC) has performed five thorough assessments of global climate change, consistently highlighting that land use change and fossil fuel burning are the principal sources of global greenhouse gas emissions, accounting for nearly 95% of total emissions [6]. Notably, land use change plays the second most important role in increasing the global atmosphere’s CO_2_ concentration, following only fossil fuel burning [7]. According to the Global Carbon Project’s 2020 estimates, the total land use carbon emissions (LUCE) between 1850 and 2019 was approximately 265 GtC [8]. A thorough analysis of the interaction between carbon emissions and land use change is necessary due to the importance of the feedback between the land system and the atmosphere [5]. Additionally, land use change has an important effect on the ecosystem by changing the spatial arrangements of local resources, biodiversity, and ecosystem types, directly affecting the spatial pattern of the ecosystem service value (ESV) [9,10]. Anthropogenic activities have increased the deterioration of terrestrial surfaces in recent years, resulting in broad land use changes that negatively influence ecosystem services and become considerable challenges to the environment. Consequently, researching the geographical variability of ESV changes caused by land use change is critical for assessing the environmental effect of human activities.

China’s increasing industrialization and urbanization have resulted in significant carbon emissions, ecological damage, and other environmental challenges [11]. To tackle climate change and environmental problems, the Chinese government has created and implemented several policies and actions [12]. For the first time, ecological civilization development was included in China’s five-year plan in 2015 [13]. It was added to the Constitution in 2018 [14]. A solemn pledge to achieve a peak level of carbon emissions by 2030 and zero carbon emissions by 2060 was made by the Chinese government in 2020 [15]. Balancing economic development and environmental protection is a major issue in China. Researching the relationship between LUCE and ESV provides important scientific evidence for solving this problem. By quantifying LUCE and ESV under different land use scenarios, policymakers can be provided with references to help them promote economic development while minimizing negative environmental impacts, thereby achieving sustainable development.

Numerous studies on LUCE have been conducted recently. These studies have primarily concentrated on carbon budget accounting of land use change [16], driving mechanisms and influencing factors of LUCE [17], and the spatial distribution of LUCE [18], etc. The carbon accounting methods primarily include the thinning model, plot inventory method, and IPCC emission factor method. Land use structure, population growth, and economic output are also significant factors contributing to the increase in LUCE. Since the publishing of the UN Millennium Ecosystem Assessment Report, there has been substantial global interest in the study and assessment of ecosystem services [19–22]. Land use and ESV are intricately connected. Current research predominantly focuses on understanding how the pattern, type, and intensity of land use impact ecosystem service functions [23,24]. Additionally, there is a growing interest in investigating the temporal and spatial characteristics of ESV in response to land use changes [25,26], conducting ESV assessments under various Land Use and Cover Change (LUCC) scenarios [27], and the development of land use patterns based on ESV [27]. Two research methods were used to evaluate ESV. The first method is based on the unit pricing of ecosystem service items, whereas the second method is based on the comparable unit area factor [28]. In the former, ecosystem processes and functions are often calculated using ecological models, and after that, ESV is estimated using direct market procedures, alternative market methods, and simulation market value approaches [29]. However, because this approach requires vast data and difficult computations, it is best suited for small-scale regions or unique ecosystems [30,31]. The latter method inputs the initial ESV per unit area to determine the overall ESV [32]. This method is generally simple, applicable to ESV evaluation in the context of land use change, and widely utilized across large regions [33]. However, most research on LUCE and ESV has been investigated individually, with little research on the spatial link between them. At the same time, these studies often employ autonomous administrative entities as research areas, which compromises the integrity of evaluating the economic, social, and ecological implications of LUCE and ESV. As a result, it is vital to investigate cross-administrative urban circles and river basins as a whole.

YRB is vital to China, acting as both an industrial zone and an ecological barrier. It is a significant source of energy and raw materials, as well as the country’s primary industrial base. The government has highly prioritized the control and development of the YRB since the formation of the People’s Republic of China. The ecological preservation and development of a high-quality YRB plan [34], which was declared an effective nationwide strategy in 2019, will be critical in developing ecological civilization and promoting high-quality economic growth in the future. Emphasizing ecological importance, low-carbon measures, and green development are acknowledged as the only realistic approaches to fulfilling the YRB’s high-quality development goals.

In summary, this paper primarily investigates the following three aspects: (1) Estimation of LUCE and ESV in 115 prefecture-level cities within the YRB. (2) Analysis of the spatiotemporal patterns of LUCE and ESV in the YRB. (3) Finally, the paper explores the spatial relationship between land use carbon emissions and ecological service value, aiming to provide references for improving ecological environment quality and reducing carbon emissions in the YRB. The remainder of the paper is structured as follows: the ’Materials and Methodology’ section, the ’Result and Discussion’ section, and the ’Conclusions’ section.

## 2. Materials and methodology

### 2.1 Theoretical analysis

Land is an important bridge between humans and nature [35]. Land use change affects ecosystem services and carbon emissions by changing land surface structure and cover. Land use change mainly includes changes in utilization mode, spatial pattern, management mode, etc. Land use type, vegetation cover, ecological pattern, landscape type, ecological efficiency, and resource distribution have an impact on the function, structure, and biological process of the ecosystem, which can be applied to ESV and ultimately determine the regional ecological environment quality. In addition, land use change affects regional net carbon emissions through carbon source/sink structure and carbon cycle system [36]. The frequent change in land use directly affects the carbon emissions of land use and human activities. Changes in land use patterns, spatial patterns, and management patterns not only affect ecosystems but also affect carbon source/sink structure and carbon cycle system balance, thus affecting net carbon emissions. Therefore, this study provides theoretical support for the spatial relationship between LUCE and ESV.

### 2.2. Study area

**Fig 1:**
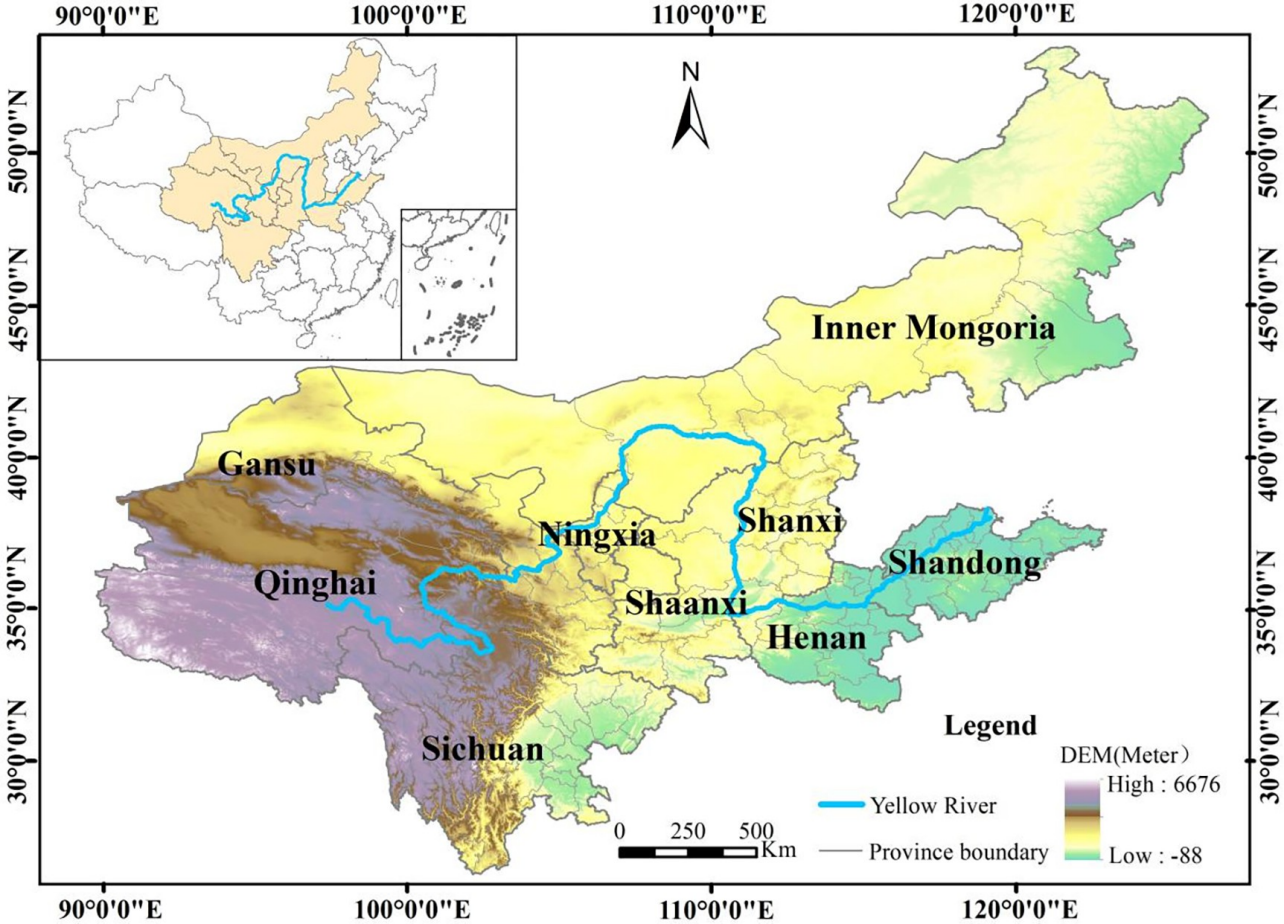
Location of the study area.

The YRB spans nine provinces [37] and includes 115 prefecture-level cities (Fig 1). The nine provinces include Inner Mongolia, Shandong, Henan, Shanxi, Shaanxi, Ningxia, Sichuan, Qinghai, and Gansu. This region plays a critical role as a key ecological barrier and economic belt, having a significant impact on Chinese economic and social growth while also maintaining environmental safety [38]. Remarkably, the combined GDP of these nine provinces reached 25,346.4 billion CNY in the year 2020, constituting 25% of the overall GDP of China. Additionally, the population of this region accounted for 15.14% of the total population of China.

### 2.3. Data sources

The land use data utilized in this study were derived from Landsat TM image interpretation data (https://www.resdc.cn/) and covered five time periods (2000, 2005, 2010, 2015, 2020) (Table 1). The data were formatted in raster, possessing a spatial resolution of 30 meters and an interpretation accuracy exceeding 90%. This dataset uses a two-tier classification system: the first tier is divided into six categories based primarily on land resources and their usage attributes, namely cropland, woodland, grassland, water, construction land, and unused land; the second tier is based mainly on the natural attributes of land resources and is divided into 23 types. In this study, the classification of land use types utilizes the six categories from the first-tier classification. The energy consumption and socioeconomic statistics were gathered from statistical sources for the relevant years, including the China Energy Statistical Yearbook (2000–2021), the Statistical Yearbook (2001–2021), and the National Agricultural Product Cost and Income Statistics Compilation (2001–2021). Because of data availability, energy consumption figures for prefecture-level cities in the YRB were calculated from 2000 to 2020 by computing the ratio of each city’s GDP within the research area to the GDP of the province in which it is located.

**Table 1:** Data sources.

Data type	Data name	Source
Land data	LUCC data (2000,2005,2010,2015,2020)	Landsat TM image interpretation data (https://www.resdc.cn/)
The energy consumption statistics	Energy consumption statistics	Energy Statistical Yearbook (2001–2021)
The socioeconomic statistics	Area and price of rice, wheat, and corn sown	National Agricultural Product Cost and Income Statistics Compilation (2001–2021)

### 2.4. Methodology

#### 2.4.1. Method for calculating LUCE

(1) Method for calculating direct LUCE

The formula for calculating LUCE is [39]:

$$E_{d}=\sum e_{i}=\sum T_{i}\times \beta_{i}$$ (1)

where *E*_*d*_ is the total direct carbon emissions; *e*_*i*_ represents the carbon emissions generated in land use type *i*; *T*_*i*_ is the area of land use type *i*; and *β*_*i*_ is the carbon emission (absorption) coefficient of land use type *i*. The carbon emission (absorption) coefficients of cropland, woodlands, grassland, water, construction land, and unused land were calculated to be 0.422 t/ha^2^.a [40], -0.644 t/ha^2^.a [19], -0.021 t/ha^2^.a [19], -0.253 t/ha^2^.a [40], and -0.005 t/ha^2^.a [40], respectively.

(2) Method for calculating indirect LUCE

The indirect estimation method was used to calculate the carbon emissions of construction land, which was estimated using consumption and carbon emission coefficients of various energy sources in conjunction with the China Energy Statistical Yearbook and the 2006 IPCC National Greenhouse Gas Emission Inventory Guide. The following energy sources were chosen for this paper: raw coal, coke, crude oil, gasoline, kerosene, diesel oil, fuel oil, natural gas, and electricity.

The calculation formula is as follows:

$$E_{\upsilon }=\sum e_{i}=\sum E_{i}\times \eta_{i}\times \lambda_{i}$$ (2)

where *E*_*ν*_ is the carbon emission of construction land; *e*_*i*_ is the carbon emission created by the consumption of energy *i*; *E*_*i*_ is the consumption of energy *i*; *ƞ*_*i*_ is the coefficient of the consumption of energy *i* converted into standard coal; and *λ*_*i*_ is the carbon emission coefficient of energy *i*.

#### 2.4.2. Value accounting of ESV

The standard equivalent is the economic value of the annual natural grain yield of farmland with a 1 hm^2^ national average yield, and it can be used to characterize and quantify the potential contribution of different types of ecosystems to ecological service functions [41]. The approach of Xie [42] was used to calculate the standard equivalent of ecosystem service value as the net profit of grain production per unit area, and the calculation formula was as follows:

$$D=S_{r}\times F_{r}+S_{w}\times F_{w}+S_{c}{\times F}_{c}$$ (3)

where *D* is the ESV of one standard equivalent factor (CNY/hm^2^); *S*_*r*_, *S*_*w*_, *and Sc* are the percentages (%) of rice, wheat, and corn sown area in the country to the total sown area of the three crops, respectively; and *F*_*r*_, *F*_*w*_, *F*_*c*_ represent the national average net profit per unit area of rice, wheat and corn, respectively (CNY/hm^2^). Data on the average proportion of rice, wheat, and maize seeded and the average net profit per unit area in China from 2000 to 2020 were used to describe and quantify the potential contribution of different ecosystem types to ecological services. All price values for the years have been adjusted to real values based on the year 2000 as the base year. Eq (3) yields a D value of 2163.71 CNY/hm^2^.

ESV is calculated using the following formula:

$$ESV=\sum ESV_{t}$$ (4)

$$ESV_{t}=\sum (A_{t}\times VC_{ti})$$ (5)

$$VC_{ti}=ES_{ti}\times D$$ (6)

where *ESV* is the value of the ecosystem services, *ESV*_*t*_ is the value of the ecosystem services for ecosystem type *t*, *A*_*t*_ is the area of ecosystem type *t* in the study area (hm^2^), *VC*_*ti*_ is the value coefficient of the *i* ecological service of the t ecosystem type in the study area (CNY/hm^2^), and *ES*_*ti*_ is the equivalent value of *i* ecological service function of the t ecosystem type. The ESV coefficients for each land use type are listed in Table 2, and the ESV of the construction land was ignored.

**Table 2:** ESV coefficients in the YRB for each land-use type (CNY/hm^2^a).

First category	Subcategories	Cropland	Woodland	Grassland	Water	Unused land
Provisioning service	Food production	2164	714	930	1147	43
	Raw materials	844	6448	779	757	87
Regulating service	Gas regulation	1558	9347	3246	1103	130
	Climate regulation	2099	8806	3375	4457	281
	Hydrological regulation	1666	8850	3289	40613	151
Supporting service	Waste disposal	3008	3722	2856	32131	563
	Soil conservation	3181	8698	4847	887	368
	Biodiversity maintenance	2207	9758	4046	7422	865
Cultural service	Landscape aesthetics	368	4501	1882	9607	519
	Total	17093	60843	25250	98124	3008

#### 2.4.3. Spatial autocorrelation analysis

(1) Global spatial autocorrelation analysis
$$I=\frac{n\sum_{i=1}^{n}\sum_{j=1}^{n}w_{ij}(x_{i}-\bar{x})(x_{j}-\bar{x})}{\sum_{i=1}^{n}\sum_{j=1}^{n}w_{ij}\sum_{i=1}^{n}{\left(x_{i}-\bar{x}\right)}^{2}}$$ (7)
  where *I* represents the global Moran’s value; n represents the number of individuals; *x*_*i*_ and *x*_*j*_ denote the observation on the study object; *w*_*ij*_ is the spatial contiguity weights matrix of the research objects *i* and *j*; and $\bar{x}$ is the average value of the attribute value of the research object.
$$Z(I)=\frac{1-E(I)}{\sqrt{VAR(I)}}$$ (8)
  where *E(I)* is the observed variable’s autocorrelation expectation *and VAR(I)* is the variance value. When |*Z(I*)|>1.96, the significance level is less than 5%; when *|Z(I*)|>2.58, the significance level is 1%.
(2) Local spatial autocorrelation analysis
  Local spatial autocorrelation (LISA) is a technique that uses local Moran’s I to highlight the agglomeration of various factors in specific areas within the research region. The LISA index may be used to divide global Moran’s I into different sections of the full spatial scope, as shown by the expression:
$$I_{i}=x_{i}^{\prime }\sum_{j=1}^{n}w_{ij}x_{j}^{\prime }$$ (9)
  where *x’*_*i*_ and *x’*_*j*_ are the standardized unit observation values, and *w*_*ij*_ is the weight, which is the same as global spatial autocorrelation.
(3) Bivariate spatial autocorrelation analysis
  Bivariate spatial correlation can be utilized to evaluate the spatial autocorrelation level of two variables, and this method is used to establish whether there is a spatial connection between two variables. This research employed two versions of bivariate Moran’s I: global and local. For the global bivariate Moran, I analyzed the existence and degree of spatial relationship between LUCE and ESV throughout the whole region, whereas for the local bivariate Moran, I studied the spatial correlation at a single location. The calculation formulas are as follows:
$$I=\frac{\sum_{i=1}^{n}\sum_{j=1}^{n}w_{ij}(x_{i}-\bar{x})(x_{j}-\bar{x})}{S^{2}\sum_{i=1}^{n}\sum_{j=1}^{n}w_{ij}}$$ (11)
$$I_{i}=\frac{(x_{i}-\bar{x})\sum_{j=1}^{n}w_{ij}(x_{i}-\bar{x})}{S^{2}}$$ (12)
  where *I* is the global bivariate *Moran’s I*; *S*^*2*^ is the variance; and *Ii* is the local bivariate *Moran’s I*.

## 3. Result and discussion

### 3.1. Land use change from 2000 to 2020

**Fig 2:**
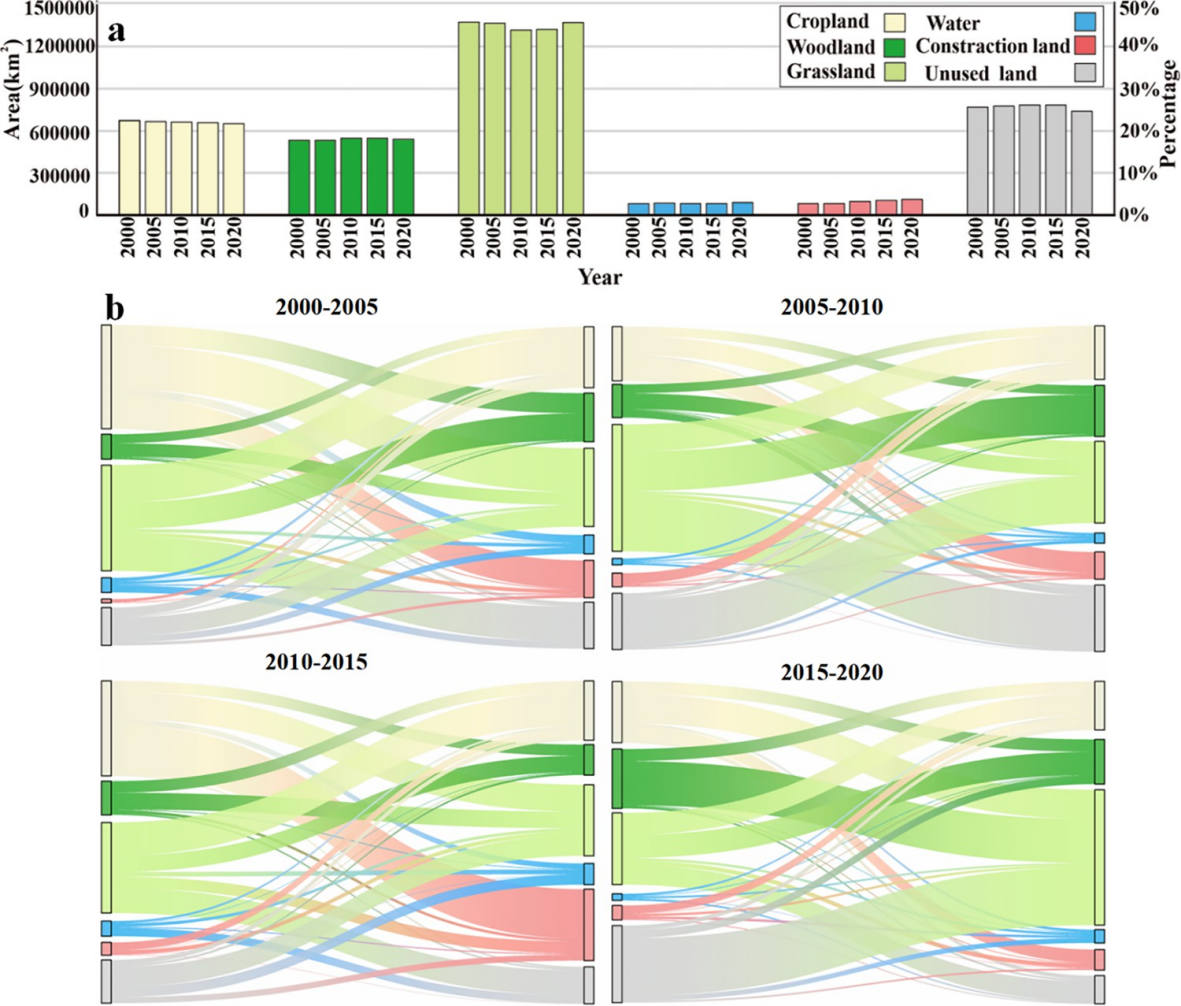
Land use change from 2000–2020 in the YRB. (a. Changes in area and proportion of six land use types from 2000 to 2020. (b. Sankey map of land use transfer matrix).

Throughout the research period, the area and change in each land use type were quite different (Fig 2A). Grassland, unused land, farmland, and woodlands were dominant in the YRB. From 2000 to 2020, the construction land area increased significantly, increasing by 2.94×10^4^ km^2^, or 45.61%. Cropland land area decreased, from 6.72×10^5^ km^2^ in 2000 to 6.50×10^5^ km^2^ in 2020. Throughout the research period, the areas of woodland, grassland, and water increased; furthermore, the water area increased the most, while the unused land area decreased.

The land use transfer matrix (Fig 2B and Table 3) shows that cropland was the principal source of the growth in construction land. From 2000 to 2020, approximately 3.15×10^4^ km^2^ of cropland was converted to construction land, demonstrating that the YRB’s increase in urbanization has come at the price of farming. Woodland has primarily been converted to grassland, while water areas and construction land have primarily been converted to cropland. Furthermore, unused land has been turned mostly into grassland. The conversion of construction land to agricultural land may be linked to rapid urban growth, resulting in a considerable inflow of rural dwellers into towns. Consequently, the reduced demand for construction land in rural areas has facilitated the conversion of such land to cropland.

**Table 3:** Transfer matrix for land use from 2000 to 2020 (unit:km^2^).

Land use types		2020						Total area of land lost
		Cropland	Woodland	Grassland	Water	Construction land	Unused land	
2000	Cropland	—	12951	20523	4201	31570	1003	70247
	Woodland	10383	—	13633	627	1476	751	26870
	Grassland	21947	20706	—	3518	6490	15941	68602
	water	2418	270	1148	—	653	1827	6315
	Construction land	10096	247	540	1956	—	89	12928
	Unused land	3789	985	37580	6742	2185	—	51280
The total area of added		48632	35159	73425	17043	42373	19610	236242

### 3.2. Analysis of LUCE

#### 3.2.1. Time evolution characteristics of LUCE

**Fig 3:**
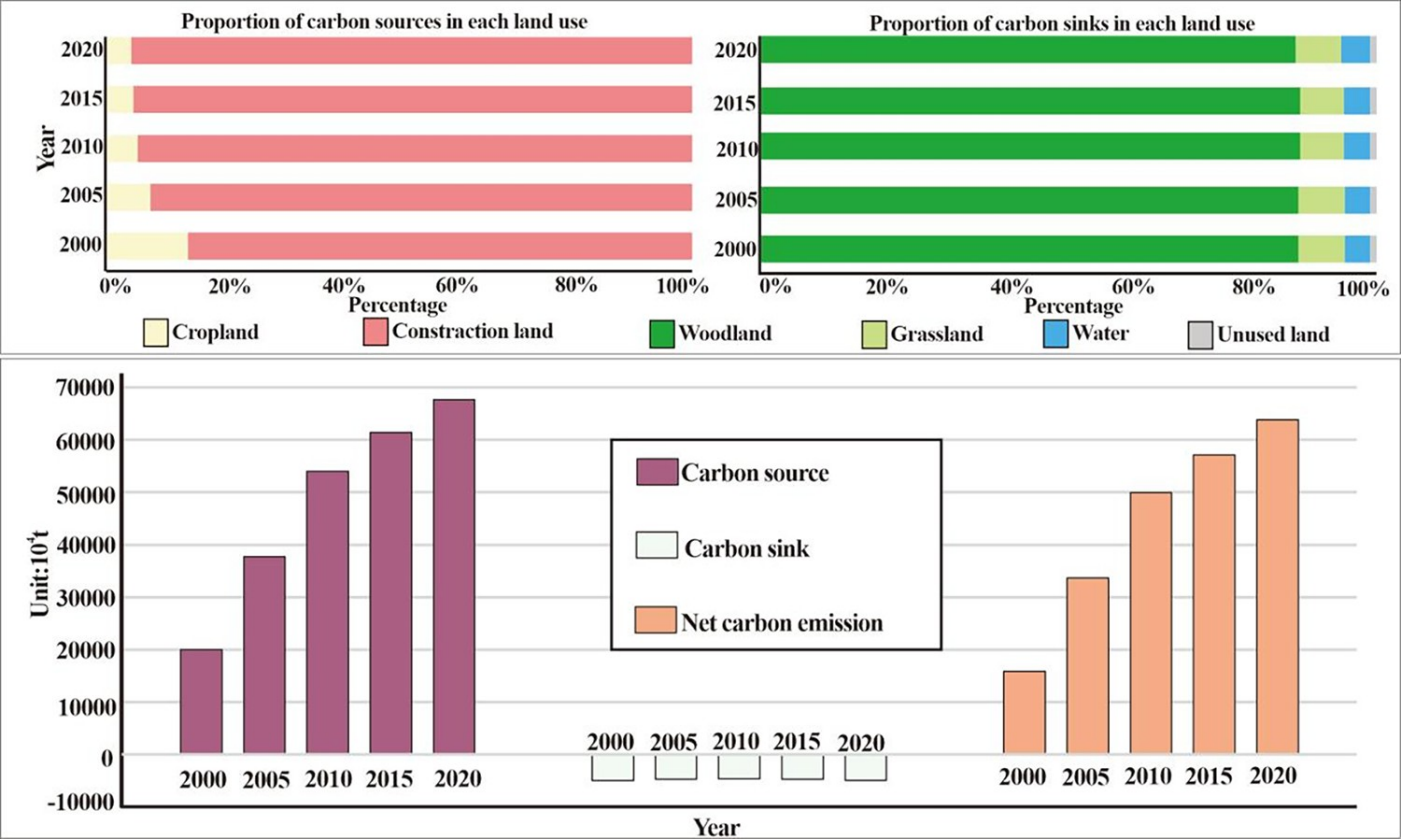
LUCE in the YRB from 2000 to 2020.

According to Fig 3, the LUCE (sum of carbon source and carbon sink) is increasing in the YRB, with the total LUCE increasing from 1.68×10^8^ tC/year in 2000 to 6.42×10^8^ tC/year in 2020. The trend of this change remains consistent with the previous research [43]. Construction land was the principal source of LUCE. Carbon sources from construction land increased from 89.75% to 96.75% from 2000 to 2020, whereas carbon sources from croplands decreased from 10.25% to 3.25% (Fig 3). Carbon sinks played a key role in sequestering LUCE in the region, with woodland being the most significant contributor, accounting for over 86.93% of the total carbon sinks. It was followed by grassland, water, and unused land, accounting for approximately 13.07% of the total. From 2000 to 2020, the overall carbon sink capacity in the YRB exhibited minimal fluctuations, experiencing an initial increase followed by a subsequent decrease. The aggregate annual carbon sink capacity is generally maintained at 7.87×10^7^ tC/year. However, the offsetting contribution from cropland and construction land sources decreased from 28.66% in 2000 to 8.14% in 2020.

#### 3.2.2. Spatial evolution characteristics of LUCE in the YRB

**Fig 4:**
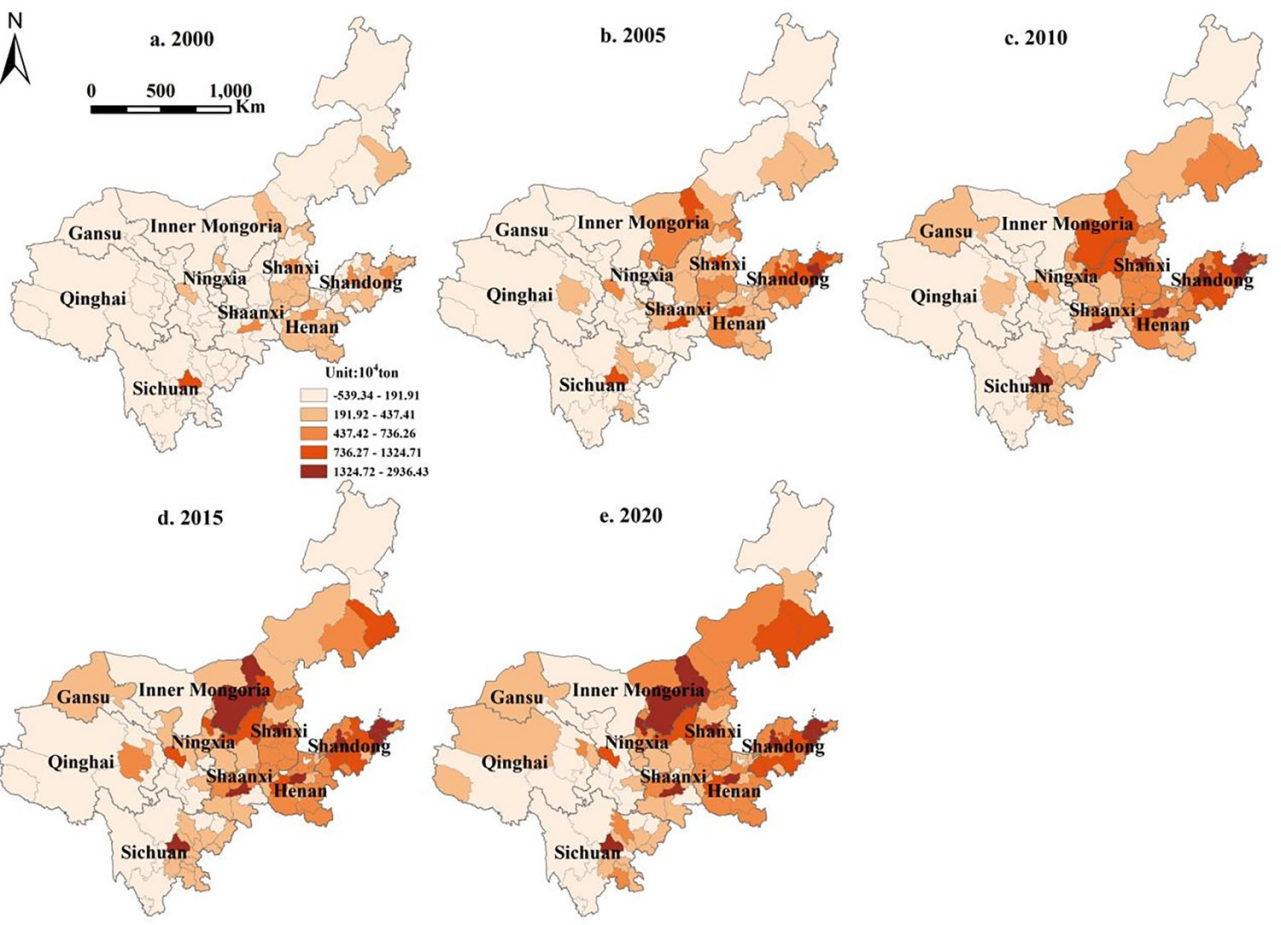
Map of LUCE in the YRB.

The spatiotemporal dynamics of LUCE in the YRB are depicted in Fig 4 and show a steadily increasing trend in total LUCE. The distribution pattern revealed that emissions were larger in both the middle and the lower reaches, while they were lower in the upper reaches. Cities with a high LUCE were concentrated mostly in Shandong, Henan, Shanxi, and Inner Mongolia, with a minor concentration noted in other provinces’ capital cities. Notably, Shandong and Henan Provinces have emerged as key energy-intensive regions with increasing urbanization and abundant construction land. Similarly, Shanxi Province and Inner Mongolia are important energy-producing provinces in China, contributing to increased LUCE. Lower LUCE zones, on the other hand, are mostly located in Qinghai Province, Gansu Province, western Sichuan Province, eastern and western Inner Mongolia, and the eastern half of Shaanxi Province. These places have relatively weak regional economies and slow urban growth. Concurrently, these areas have strong ecological preservation initiatives inside China. The existence of vast woodlands, grasslands, and other natural habitats increases their carbon sequestration capability, resulting in a lower LUCE.

From 2000 to 2020, the YRB’s Global Moran’s I of LUCE has a positive value (Table 4), demonstrating that LUCE are positive spatial autocorrelation in the YRB. Previous studies on LUCE have also reached consistent conclusions [19,44,45]. Simultaneously, Z> 2.58 implies that the distribution of LUCE exhibits aggregation characteristics similar to the spatial positive correlation model, showing that cities in the YRB with greater or lower LUCE tend to cluster together in space. This clustering might be related to economic disparities [46]. The closeness in industrial structure and economic growth model across nearby cities may contribute to their comparable economic scale and development [19]. Furthermore, the economic growth of urban industries is likely connected with energy consumption, and surrounding cities frequently demonstrate significant similarity in their economic development [47].

**Table 4:** Global Moran’s I of LUCE.

Year	2000	2005	2010	2015	2020
Moran’s I	0.280	0.407	0.344	0.272	0.235
Z	4.837	6.869	5.867	4.721	4.123

**Fig 5:**
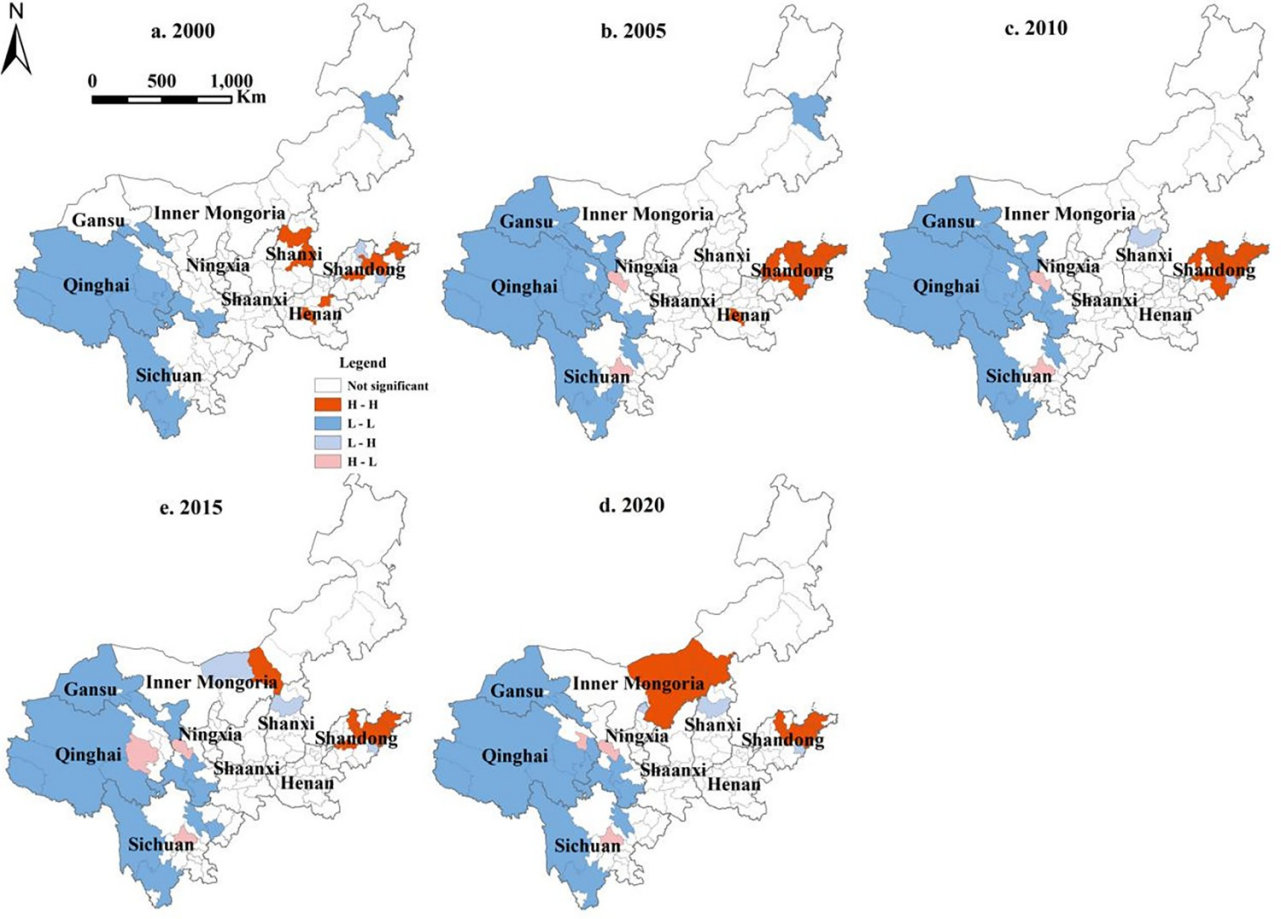
LISA aggregate map of LUCE in the YRB.

In the analysis of local spatial autocorrelation, LUCE at the city level in the YRB exhibits four types of spatial autocorrelation: High-High agglomeration (H-H), Low-Low agglomeration (L-L), High-Low agglomeration (H-L), and Low-High agglomeration (L-H). The spatial agglomeration change in the total LUCE in the YRB was not significant (Fig 5); there were features of H-H and L-L and a sporadic distribution of H-L and L-H type cities. The H-H-type cities were found primarily in Shandong, Henan, Inner Mongolia, and Shanxi. Shandong and Henan are large economic provinces in China with high economic generation levels, high energy consumption, and high urbanization rates, resulting in substantial carbon emissions. Shanxi and Inner Mongolia are major energy provinces in China, and their economic development is heavily reliant on energy, resulting in significant carbon emissions. In terms of the temporal scale, Shanxi, Henan, and Shandong exhibited the highest number of H-H-type cities in 2000. By 2005, only Henan and Shandong still had H-H cities, while Shandong Province witnessed a steady expansion in their count. In 2010, H-H-type cities were observed exclusively in Shandong Province, and their prevalence was comparable to that in Shandong Province in 2005. Subsequently, in 2015, H-H-type cities emerged in both Shandong Province and Inner Mongolia, accompanied by a decrease in the number of such cities in Shandong Province. By 2020, H-H-type cities were once again concentrated solely in Shandong Province and Inner Mongolia, resembling the pattern observed in 2015. However, the number of H-H-type cities in Shandong Province declined, whereas the number of H-H-type cities in Inner Mongolia increased. These changes were because China’s national socioeconomic development in 2000, for the first time in the tenth five-year plan, stated the country would "actively and steadily promote urbanization" as one of the focuses of the national development strategy [48], 16th National Congress of the Communist Party of China in 2002 [49], and further put forward "the rural economy flourish and speed up urbanization," at this point in China’s rapid urbanization process. As the sole "river-sea intersection area" along the Yellow River and coastal areas, Shandong Province has clear geographical advantages [50]. It has had the quickest economic development and urbanization rate of the nine provinces in the YRB. However, economic progress and urban expansion necessitate significant energy use and construction land, resulting in significantly high carbon emissions. As a result, H-H cities were concentrated in Shandong Province from 2000 to 2010. Shandong has made significant efforts to alter the industry structure, energy, transportation, agricultural input, and land usage since the foundation of ecological civilization in 2012 [51], therefore limiting the growth rate of LUCE and the number of H-H-type cities. Coal consumption accounts for up to 80% of Inner Mongolia’s energy structure as its core industry [52]. Inner Mongolia’s industrial structure has long been dominated by the iron and steel, coal chemical, thermal power, and nonferrous metal sectors. The expansion of these sectors is the primary cause of the growth in regional coal consumption in Inner Mongolia. Coal power consumes 50% to 60% of total local coal use all year [53]. From 2012 to 2021, Inner Mongolia built China’s largest coal power and coal chemical base [54], resulting in massive amounts of emissions of carbon dioxide. The energy structure is relatively simple, but the industrial transformation is complex, resulting in a continuing concentration of H-H cities in Inner Mongolia. The overall distribution of L-L cities was relatively stable, with most of them found in Qinghai, Gansu, and Sichuan, as well as in the eastern section of Inner Mongolia. Because woodlands and grassland cover a large fraction of these regions, the economy is comparatively underdeveloped, the construction land area is small, and the overall LUCE is low. The H-L city distribution was reasonably consistent and distributed around the H-H city distribution. H-L type cities were found mostly in western provincial capital cities in Qinghai and Sichuan, which are near L-L type cities and have a positive spillover impact on them.

### 3.3. ESV analysis

#### 3.3.1. ESV analysis from various land use types

The total ESV in the YRB increased from 8.7037.45×10^8^ CNY to 88187.38×10^8^ CNY from 2000 to 2020 (Table 5). The entire performance of ESV in the YRB increased, decreased, and increased again, showing an overall increasing tendency. The overall trend of change aligns with the findings of previous research [55]. The ESV in the YRB increased from 2000 to 2010, which may be ascribed to China’s 2002 policy of restoring cropland to woodland. Sichuan, Shaanxi, and Gansu began piloting the policy overhaul in 1999. As a result, the scale of woodland in the YRB increased significantly throughout this period, thereby increasing the ESV. The decline in ESV between 2010 and 2015 can be attributed to an improvement in the overall socioeconomic level and the acceleration of urban development, which resulted in a significant expansion of land for housing and infrastructure construction. The expansion of construction land caused a reduction in the areas of arable land and grassland, which caused a decline in ESV [55]. The ESV increased even more from 2015 to 2020, probably because ecological civilization development was included in China’s 14th Five-Year Plan in 2015 [56]. In 2018, it was also incorporated into the Constitution [57]. The growth in woodland, grassland, and water areas in the YRB has boosted the ESV as an ecological priority and green development modes have changed. The ESV of the YRB was greater upstream and lower downstream, showing a positive association. Grassland and woodland contributed the most to the overall ESV, accounting for over 38.72% and 36.70% of the ESV of each land type, respectively. Cropland ESV decreased consistently, and the cropland ESV fraction decreased from 13.20% to 12.60%. The ESV of water increased continually, and the proportion of ESV of water increased from 7.07% to 8.17%. Overall, the ESV of woodland and grassland increased, while the ESV of unoccupied land decreased, which may be connected to Northwest China’s ecological and environmental governance regulations. Many underused places, such as certain deserts, were converted into grassland or woodland.

**Table 5:** ESV statistics of land use types in the YRB from 2000 to 2020.

Year	Type	Cropland	Woodland	Grassland	Water	Construction land	Unused land	Total
2000	ESV/10^8^ CNY	11492.09	31942.04	35118.09	6157.35	0.00	2327.87	87037.45
	proportion of ESV	13.20%	36.70%	40.35%	7.07%	0.00%	2.67%	100%
2005	ESV/10^8^CNY	11382.64	32158.75	35013.95	6217.22	0.00	2331.79	87104.35
	proportion of ESV	13.07%	36.92%	40.20%	7.14%	0.00%	2.68%	100%
2010	ESV/10^8^CNY	11370.73	33241.25	33893.30	6592.58	0.00	2362.24	87460.10
	proportion of ESV	13.00%	38.01%	38.75%	7.54%	0.00%	2.70%	100%
2015	ESV/10^8^CNY	11310.70	33219.27	33831.67	6650.94	0.00	2360.30	87372.88
	proportion of ESV	12.95%	38.02%	38.72%	7.61%	0.00%	2.70%	100%
2020	ESV/10^8^CNY	11114.99	32423.55	35215.93	7201.05	0.00	2231.86	88187.38
	proportion of ESV	12.60%	36.77%	39.93%	8.17%	0.00%	2.53%	100%

#### 3.3.2. ESV spatiotemporal characterization in the YRB

**Fig 6:**
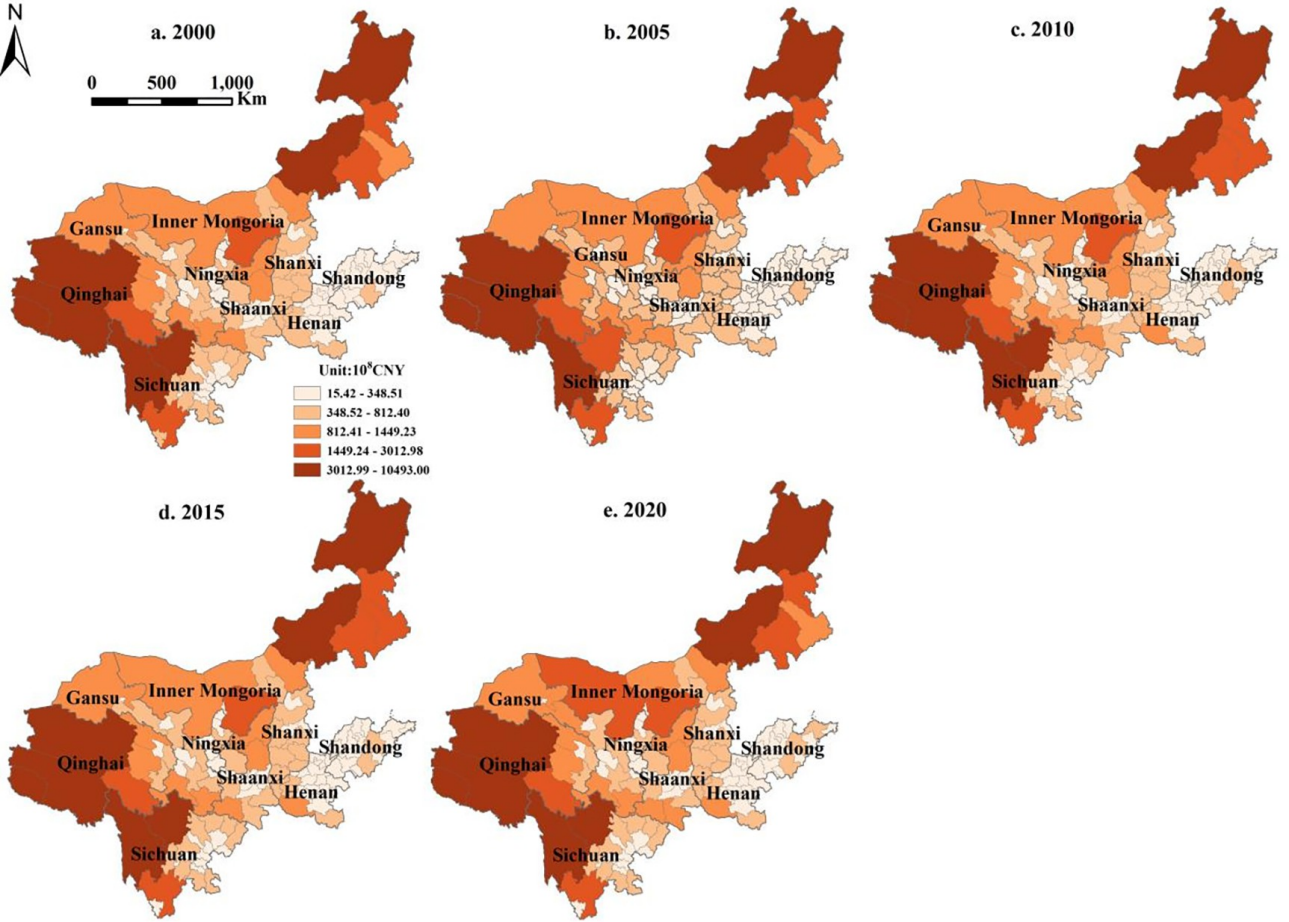
ESV map in the YRB.

Fig 6 depicts the spatiotemporal pattern of the ESV, indicating a relatively stable spatial distribution from 2000 to 2020. Overall, the upstream regions exhibited higher ESV values than the downstream regions, which formed the opposite characteristic of the spatial distribution of LUCE. High ESV cities were concentrated in Qinghai Province, western Sichuan Province, and northeastern Inner Mongolia, whereas low ESV cities were concentrated in Henan, Shanxi, and central Shaanxi. These locations have relatively undeveloped economies, less construction land and cropland areas, and more woodland and grassland areas, all of which contribute to their high ecological value.

Moran’s I was positive from 2000 to 2020 (Table 6), showing that ESV was positively spatially autocorrelated in the YRB. The consistent findings in previous studies also support this conclusion [58–60]. Simultaneously, Z>2.58 indicated that the ESV distribution exhibited aggregation features with the spatial positive correlation model.

**Table 6:** Global Moran’s I of ESV in the YRB.

Year	2000	2005	2010	2015	2020
Moran’s I	0.336	0.335	0.367	0.367	0.342
Z	6.2417	6.2348	6.6880	6.6808	6.325

**Fig 7:**
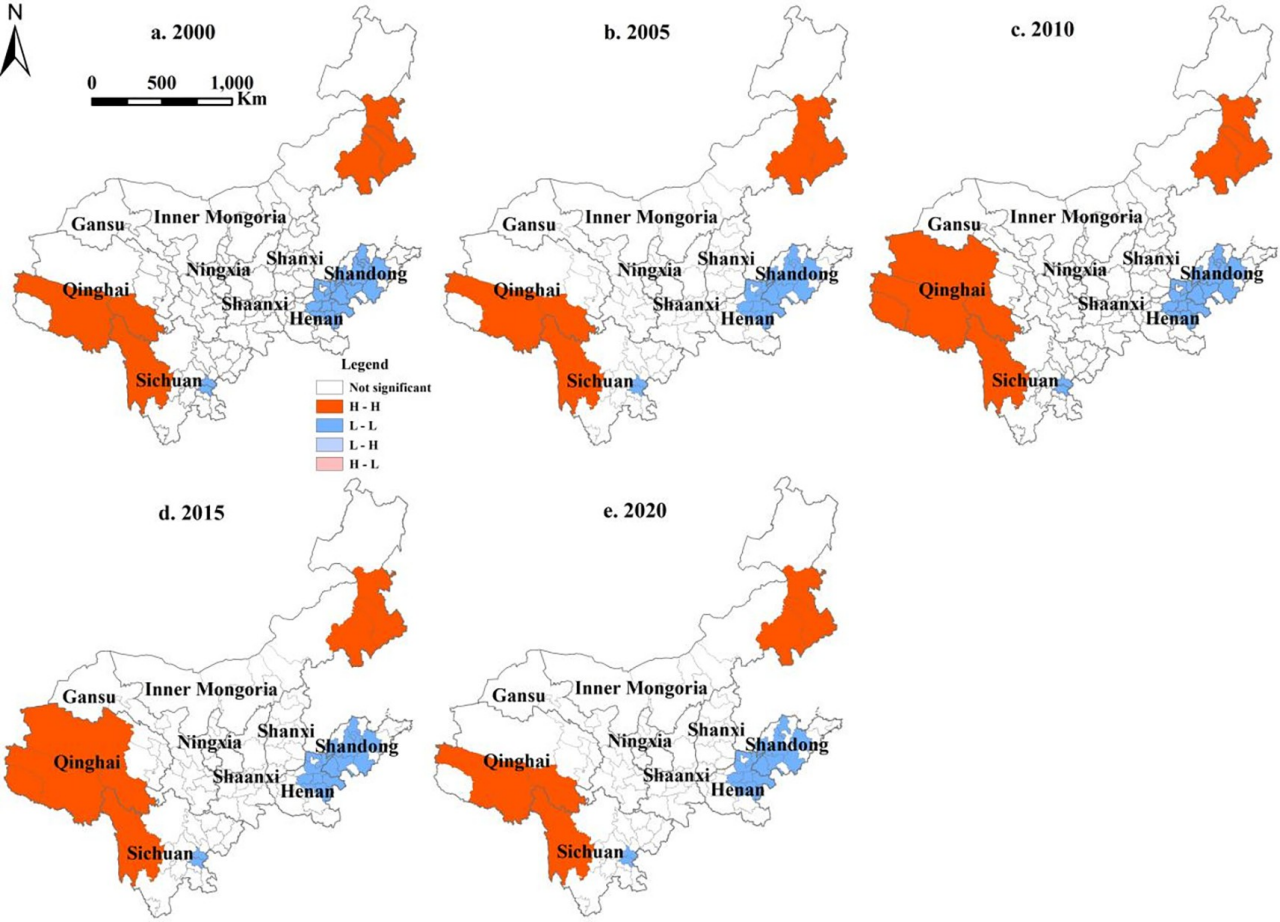
LISA aggregation map of ESV in the YRB.

According to the local spatial autocorrelation analysis (Fig 7), there were only two types of city agglomeration types: H-H and L-L; the H-H type cities were distributed primarily in Qinghai Province and western Sichuan Province, while in the eastern Inner Mongolia region, where the economy was relatively backward and construction land was scarce, had an expansive distribution. Woodland, grassland, and human activity were slightly lower; hence, they had a high regional ecological value. L-L-type cities were distributed mainly in Henan Province and Shandong Province. These areas have flat terrain, convenient transportation, and a developed economy. The large-scale increase in construction land decreased the ESV [61].

### 3.4. Spatial relationship between LUCE and ESV in the YRB

Taking into account that woodland, grassland, water, and unused land are calculated as carbon sinks due to their carbon storage capacity, and that this aspect is already included in the calculation of land use ecosystem service values, we aim to eliminate any potential bias this might introduce to the research results. Therefore, this study focuses solely on the carbon emissions from cropland and construction land when examining the spatial relationship between LUCE and ESV.

A bivariate spatial autocorrelation analysis was conducted between LUCE and ESV in the YRB (Table 7). Moran’s I was negative each year, and the absolute magnitude of the Z value was greater than 1.96, indicating a negative spatial relationship between LUCE and ESV in the research region. According to the findings, an increase in LUCE might have a detrimental influence on ecosystem values.

**Table 7:** Bivariate global Moran’s I of LUCE and ESV in the YRB.

Year	2000	2005	2010	2015	2020
Moran’s I	-0.145	-0.174	-0.157	-0.122	-0.086
Z	-3.1309	-3.7717	-3.4755	-2.6364	-1.9990

**Fig 8:**
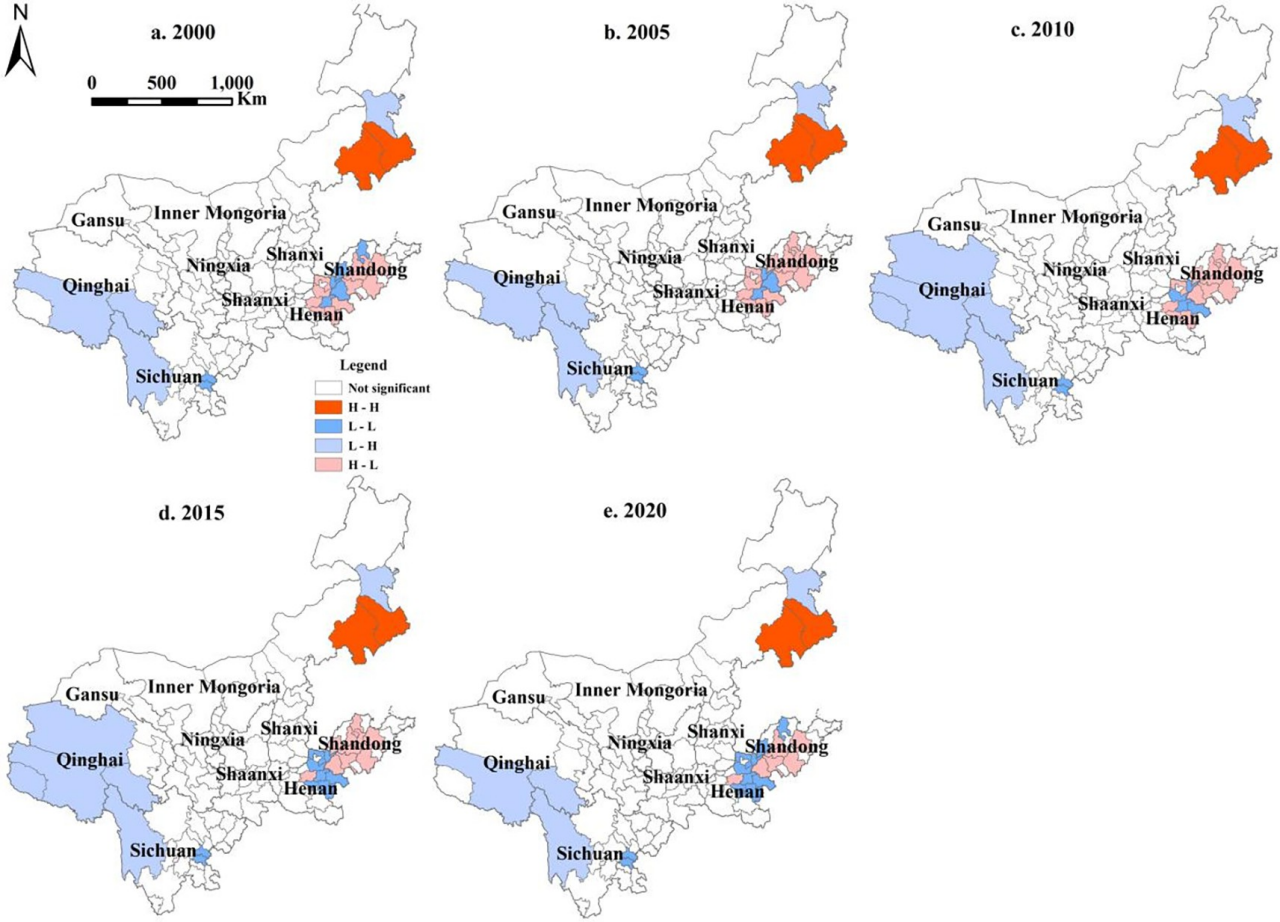
YRB LISA aggregate map of LUCE and ESV.

The findings of the local spatial autocorrelation analysis are presented in Fig 8. The regions exhibiting significant local correlation between LUCE and ESV were located primarily in Qinghai, Sichuan, Inner Mongolia, Henan, and Shandong within the YRB. Furthermore, the spatial distribution of these relationships was rather persistent. H-H cities were located mainly in eastern Inner Mongolia, whereas L-L cities were concentrated in Sichuan, Henan and select cities in Shandong. Notably, two cities located within Sichuan Province consistently maintained their L-L type classification, while L-L type cities in Henan Province and Shandong Province experienced fluctuations during the research period. H-L-type cities, however, were predominantly located in Qinghai Province as well as in Inner Mongolia. H-L-type cities were found mostly in the western section of Shandong and the northeastern region of Henan, and their classification fluctuated over the research period.

These H-H-type cities were located in eastern Inner Mongolia, indicating that these regions had high LUCE and ESV. As a result, additional increases in LUCE in this region may raise the risk posed to ESV. In the future, we must minimize LUCE, alter the industrial structure, and limit the rise of construction land. L-L cities may be found in Sichuan, Henan, and Shandong Provinces, including Neijiang and Ziyang in Sichuan. These areas had a lower LUCE and a low ESV; this result is because these areas have more cropland and less woodland and grassland than other areas. Cropland is a primary source of carbon, but its influence is far less than the right to use construction land [62]. As a result, these regions exhibited a lower LUCE. In these areas, cropland conversion to construction land should be reduced. Moreover, the area of woodland should be appropriately increased, thus enabling the area to reduce its carbon and increase its ESV. The H-L cities were distributed in Qinghai and Inner Mongolia; these areas had a low LUCE and a high ESV and were primarily composed of woodland, grassland, and cropland, with a smaller scale of construction land. Therefore, these regions not only had low LUCE and strong carbon sequestration ability but also possessed high ESV. These areas should promote environmental conservation and the growth of green and low-carbon enterprises and be regarded as priority locations for ecological compensation. H-L cities were primarily located in Henan and Shandong Provinces, where they had a high LUCE and a low ESV. These regions should speed up low-carbon production technology, increase the intensity of construction land, and reduce LUCE.

### 3.5. Policy recommendations

Based on the above research findings, the following policy recommendations are proposed:: Firstly, in high-LUCE and high-ESV (H-H type) cities such as eastern Inner Mongolia, the government should implement policies to limit new construction land development and strictly control urban expansion. This can be achieved by increasing building density, improving land use efficiency, and reducing encroachment on natural areas. Secondly, in L-L-type areas such as Sichuan, Henan, and Shandong, the government should implement measures to prevent the conversion of cropland to construction land. For example, by providing agricultural subsidies, technical support, and training, promoting sustainable agricultural technologies and practices, reducing agricultural carbon emissions, and enhancing land carbon sequestration capacity. Additionally, in areas with low LUCE and high ESV, such as Qinghai and Inner Mongolia, the government should increase investment in forest and grassland protection and restoration. Large-scale afforestation projects, restoration of degraded grasslands, and legislative protection of existing forest and grassland resources can be implemented. Simultaneously, in H-L-type cities such as Henan and Shandong, the government should vigorously promote the development of low-carbon and green industries. Through tax incentives, financial subsidies, and technical support, enterprises can be encouraged to adopt clean energy and low-carbon technologies, reduce industrial carbon emissions, and guide the transformation and upgrading of traditional industries.

Finally, in areas with high ESV and low LUCE, such as Qinghai and Inner Mongolia, the government should establish an ecological compensation mechanism, providing financial subsidies and rewards to these regions, encouraging local governments and communities to carry out ecological protection and restoration projects, and using carbon trading mechanisms to convert the carbon sequestration capacity of these areas into economic benefits. By implementing these specific policy measures, the spatial relationship between LUCE and ESV can be more effectively managed, achieving sustainable land use development and enhancing ecosystem service value.

### 3.6 Limitations and future research

Although this study met the desired research aims, it nevertheless has certain shortcomings. Due to the lack of energy data at the prefectural level, this paper calculated the energy consumption of prefecture-level cities in the YRB through the GDP ratio to calculate the carbon emissions of construction land, and errors may be introduced in the calculation results. This is because using the GDP ratio as an indirect indicator, although it can reflect the impact of economic activities on energy consumption to a certain extent, it cannot take into account factors such as region-specific energy utilization patterns, industrial structure, and technological level. Therefore, the energy consumption estimation method based on the GDP ratio may introduce estimation errors, which will affect the final carbon emission of construction land.However, considering the positive correlation between energy consumption and GDP [63], regional LUCE temporal and spatial analyses should not be affected. Thus, future research should focus on a more accurate measurement of LUCE. At the same time, the future research focus will also involve exploring how to manage the utilization of land in terms of carbon emissions and ecosystem services across administrative regions.

## 4. Conclusions

This study, taking the YRB as the research object, revealed the LUCE and spatiotemporal evolution characteristics of ESV and analyzed the spatial relationship of both in the YRB, reaching the following conclusions:

1. The YRB’s LUCE has continuously increased, with construction land acting as the dominant carbon source and woodland acting as the main carbon sink. The LUCE in the YRB had a positive spatial autocorrelation. The economically developed provinces of Henan and Shandong, along with Inner Mongolia, a major energy-producing region, constitute the concentrated area of H-H cities. Conversely, the economically disadvantaged provinces of Gansu, Qinghai, Inner Mongolia, and Sichuan form the concentrated area of L-L cities. The H-L city distribution was fairly stable and distributed around the H-H city distribution. The H-L-type cities were located mostly in the capital cities of western provinces, which were around L-L-type cities.
2. The YRB’s ESV increased. Spatially, the ESV in the YRB showed a positive autocorrelation. Only H-H and L-L-type cities had a relatively stable distribution. The distribution characteristics of H-H and L-L cities are opposite to the distribution of LUCE.
3. The LUCE was negatively correlated with ESV in the YRB, demonstrating a spatial agglomeration impact and stable distribution. Eastern Inner Mongolia had an H-H city distribution. Cities of L-L distribution in Sichuan, Henan, and Shandong Provinces, cities of H-L distribution in Qinghai Province, and Inner Mongolia, and cities of H-L distribution in Henan and Shandong Provinces.

## Supporting information

S1 File Data sources. (DOCX)
