# Supplementary material for: Spatial relationship between carbon emissions and ecosystem service value based on land use: A case study of the Yellow River Basin
Source: PLoS One. 2025 Feb 21;20(2):e0318855. doi: 10.1371/journal.pone.0318855 (PMC11845033; doi:10.1371/journal.pone.0318855)
Supplement: S1 File — (DOCX) [file pone.0318855.s001.docx]

**Data sources**

All data are obtained from publicly available sources.The land use data utilized in this study were derived from Landsat TM image interpretation data (https://www.resdc.cn/) and covered five time periods (2000, 2005, 2010, 2015, 2020)（Table 1）. The energy consumption and socioeconomic statistics were gathered from statistical sources for the relevant years, including the China Energy Statistical Yearbook (2000-2021), the Statistical Yearbook (2001-2021), and the National Agricultural Product Cost and Income Statistics Compilation (2001-2021). Because of data availability, energy consumption figures for prefecture-level cities in the YRB were calculated from 2000 to 2020 by computing the ratio of each city's GDP within the research area to the GDP of the province in which it is located.

Table 1 Data sources

| Data type | Data name | Source |
| --- | --- | --- |
| Land data | LUCC data (2000,2005,2010,2015,2020) | Landsat TM image interpretation data (https://www.resdc.cn/) |
| The energy consumption statistics | Energy consumption statistics | Energy Statistical Yearbook (2001-2021) |
| The socioeconomic statistics | Area and price of rice, wheat, and corn sown | National Agricultural Product Cost and Income Statistics Compilation (2001-2021) |
